# The Safety of Ultrasound-Guided Needle Approaches for Patellar Tendinopathy: A Theoretical Cadaveric Model

**DOI:** 10.3390/jfmk10020208

**Published:** 2025-06-03

**Authors:** Laura Calderón-Díez, Pedro Belón-Pérez, César Fernández-de-las-Peñas, José L. Sánchez-Sánchez

**Affiliations:** 1 Department of Anatomy and Histology, Faculty of Medicine, Universidad de Salamanca, 37007 Salamanca, Spain; lauca@usal.es; 2 Institute of Biomedical Research of Salamanca (IBSAL), 37007 Salamanca, Spain; jlsanchez@usal.es; 3 Real Madrid C.F., 28055 Madrid, Spain; pebelon@gmail.com; 4 Department of Physical Therapy, Occupational Therapy, Physical Medicine and Rehabilitation, Universidad Rey Juan Carlos (URJC), 28922 Madrid, Spain; 5 Department of Physical Therapy, Universidad de Salamanca, 37007 Salamanca, Spain

**Keywords:** patellar tendon, cadaver, infrapatellar nerve, tendinopathy, percutaneous electrolysis

## Abstract

Background: Patellar tendinopathy is a musculoskeletal pain condition capable of impairing physical or sport activities. Preliminary evidence supports the efficacy of percutaneous electrolysis (PE) in reducing pain and related disability in patients with patellar tendinopathy. Objective: This study proposes a theoretical model for the application of a percutaneous electrolysis approach targeting the deep zone of the proximal and distal parts of the patellar tendon in both human (ultrasound-guided) and fresh cadaver (not ultrasound-guided) models. Methods: A filiform solid needle was inserted from the lateral side of the patellar tendon targeting two areas: 1, the deep proximal interface of the Hoffa’s fat pad; and 2, the distal insertion of the patellar tendon at the tibial tuberosity in 10 fresh cadavers and in 10 healthy individuals. The patellar tendon, the saphenous nerve, and the infrapatellar nerve and its branches were identified by dissecting fresh cadavers to determine the anatomical trajectory of the infrapatellar nerve branches in relation to the needle. Results: The cadaveric model shows an anatomical relationship between the patellar tendon and infrapatellar nerve branches at the medial part of the knee. Infrapatellar nerve branches ran subcutaneously obliquely from the medial to the anterior and lateral parts of the knee, crossing in front of the patellar tendon. In all cadavers, the superior and inferior infrapatellar branches ran through the superior or inferior parts of the medial knee area. Only in 2/10 knees infrapatellar nerve branches reached the lateral part of the knee, specifically the superior lateral part. No neurovascular bundle of infrapatellar nerve branches was pierced in any insertion when the needle was inserted from the lateral part of the knee. Conclusion: This anatomical model supports the use of a lateral approach as a potentially safe approach to apply in needling interventions, e.g., percutaneous electrolysis for patellar tendinopathies. The infrapatellar nerve branches are vulnerable to needle procedures applied through the anteromedial side of the knee.

## 1. Introduction

Patellar tendinopathy, also known as jumper’s knee, is an overuse musculoskeletal condition characterized by pain localized in the proximal patellar tendon area and resulting in a decreased physical performance and sports level [1]. It commonly occurs in sports with significant loads involving jumping or changes in direction on hard surfaces [1]. Its incidence is approximately 14% in athletes, with a 32% occurrence rate in basketball players and up to 45% in volleyball players [2,3].

Current theories suggest that tendinopathies are a consequence of a failed tendon healing response to chronic overload leading to hypervascularization, changes in tendon structure with localized mucoid degeneration, and alterations in collagen fibers and the extracellular matrix [4]. Several hypotheses, including vascular [5] and mechanical causes [6], have been proposed for explaining its pathogenesis; however, current factors contributing to pain and disability remain poorly understood and are often controversial.

The anatomical location most vulnerable to injury and degeneration in the knee is the proximal and deep portion of the tendon insertion at the inferior pole of the patella (which is involved in 70% of cases) [7]. The International Tendinopathy Consensus (ICON) concluded that patellar tendinopathy is the preferred term for persistent patellar tendon pain and loss of function related to mechanical loading [8]. The diagnosis of patellar tendinopathy is mainly based on clinical symptoms, since imaging is not always necessary for its diagnosis [8]. Patients usually report insidious pain in the anterior knee area that is reproduced by manual palpation of the tendon. Thus, pain provocation maneuvers, such as a single-leg squat at 30º flexion, are typically positive [9].

The therapeutic approach of patellar tendinopathy is heterogeneous. Evidence suggests that exercise programs focused on tendon load modification are the treatment strategies with the highest level of evidence for this condition [10]. If symptoms persist, platelet-rich plasma injections, and minimally invasive physiotherapy techniques, e.g., dry needling or percutaneous electrolysis, are considered as therapeutic options [11,12]. Percutaneous electrolysis consists of the application of a galvanic electrical current through a solid needle. Different systematic reviews and meta-analyses have found moderate evidence showing that percutaneous electrolysis is effective for reducing pain and disability in patients with different tendinopathies, including a patellar one [13,14,15]. In fact, a secondary analysis revealed that percutaneous electrolysis exhibited better cost-effectiveness in terms of quality-adjusted life years as compared to the dry needling group [16]. Nevertheless, no consensus exists on the intensity of the galvanic electrical current needed to get the best clinical results [17]. A recent review concluded that percutaneous electrolysis generates a controlled local pro-inflammatory effect, regulates inflammation, and facilitates subsequent healing by improving extracellular matrix synthesis during the first 7 days after its application [18].

However, therapeutic interventions, particularly those involving the insertion of needles, have potential risks. For example, in the knee area, iatrogenic injury to the infrapatellar nerve, a sensory branch of the saphenous nerve composed of cutaneous branches that collect peripatellar sensitivity, has been documented. This nerve and its branches are anatomically related to medial knee structures and the patellar tendon, making it susceptible to injury during some therapeutic procedures like knee replacement, medial arthroscopic approaches, or even anterior cruciate ligament reconstructions [19,20,21]. Accordingly, this nerve may also be at risk of injury during the treatment procedures inserting a needle into the patellar tendon area from its medial side. These lesions of the saphenous nerve or its infrapatellar branches present clinically as a neuritis or postoperative neuroma with neuropathic pain and, in some cases, a loss of sensitivity or paresthesia in the medial infrapatellar region of the lower extremity [22,23]. Some cases of reflex sympathetic dystrophy of the knee have even been attributed to iatrogenic injury of infrapatellar branches of the saphenous nerve [22,23].

To prevent and reduce potential nerve injuries, some authors have proposed the use of ultrasound guidance during knee surgeries as safer procedures [24,25]. In fact, it is also recommended that the application of some conservative procedures such as extracorporeal shock waves, infiltrations, or needling therapies should be performed under ultrasound guidance. To date, different studies have analyzed the effects of ultrasound-guided percutaneous electrolysis on the patellar tendon [26,27,28,29]; however, these studies have not defined their trajectory parameters for safety technique execution, and they have not reported the presence of adverse events. A recent cadaveric study showed that the accuracy of needling insertion into the patellar tendon targeting the fat–patellar tendon interface was higher when the procedure was ultrasound-guided than when it was conducted based just on an anatomical landmark [30]. All these studies used an approach from the medial border of the patellar tendon [26,27,28,29,30]. Since the infrapatellar branches of the saphenous nerve run into the medial part of the knee, a medial to lateral needling approach could lead to nerve damage. Identification of uniform criteria regarding the path approach and relationship of the patellar tendon to surrounding anatomical sensitive structures will allow for safe needling approaches. This is highly important when targeting peritendinous soft tissues e.g., the Hoffa’s fat pad. The Hoffa’s fat pad is an area of clinical relevance as it frequently presents structural alterations [28].

This study aimed to propose a theoretical model for the application of a percutaneous electrolysis approach targeting the deep zone of the proximal and distal parts of the patellar tendon in both human (ultrasound-guided) and fresh cadaver (not ultrasound-guided) models.

## 2. Methods

### 2.1. Cadaver Enrollment

A cadaveric and human validation study was performed. For the cadaveric part, 10 lower extremities from five fresh cadavers donated to the Department of Anatomy and Histology of the University of Salamanca (Spain) were used. The cadavers were checked for the presence of any structural abnormalities that could influence the anatomical study.

### 2.2. Participant Enrollement

A total of 10 healthy volunteers were recruited for the ultrasound-guided part. Participants were recruited by local announcement at the University. Since this was a validation study, we included people without symptoms in the lower extremity and without previous lower extremity surgery. The procedure with healthy participants was performed following the Declaration of Helsinki and was approved by the Human Research Ethics Committee of the University of Salamanca (CBE-1166). The participants signed a written informed consent form before their inclusion in the study. No compensation of any type was provided for the participants.

### 2.3. The Anatomical Procedure on Fresh Cadavers

**Figure 1:**
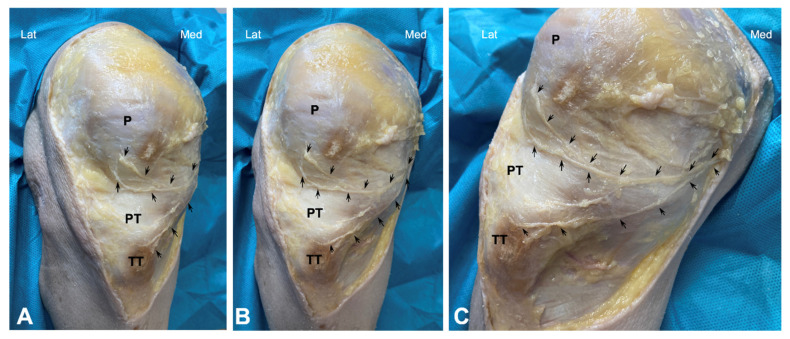
Cadaveric dissection of knee area showing the infrapatellar nerve and its branches (arrows). (P) patella, PT (Patellar tendon), and TT (Tibial Tuberosity). (**A**–**C**) show different angles.

Dissection of the anterior knee region was performed as follows: The skin and subcutaneous fatty tissue were carefully removed. A small incision was made in the fascia at the level of the sartorius muscle, and the nerves around this muscle were carefully traced by dissecting the surrounding connective tissue. This approach allowed for the identification of the saphenous nerve’s emergence. Thus, this nerve was carefully dissected distally in all cadavers to expose the main trunk as well as the infrapatellar nerve and its branches, from the medial and proximal portions to the distal portion (Figure 1).

**Figure 2:**
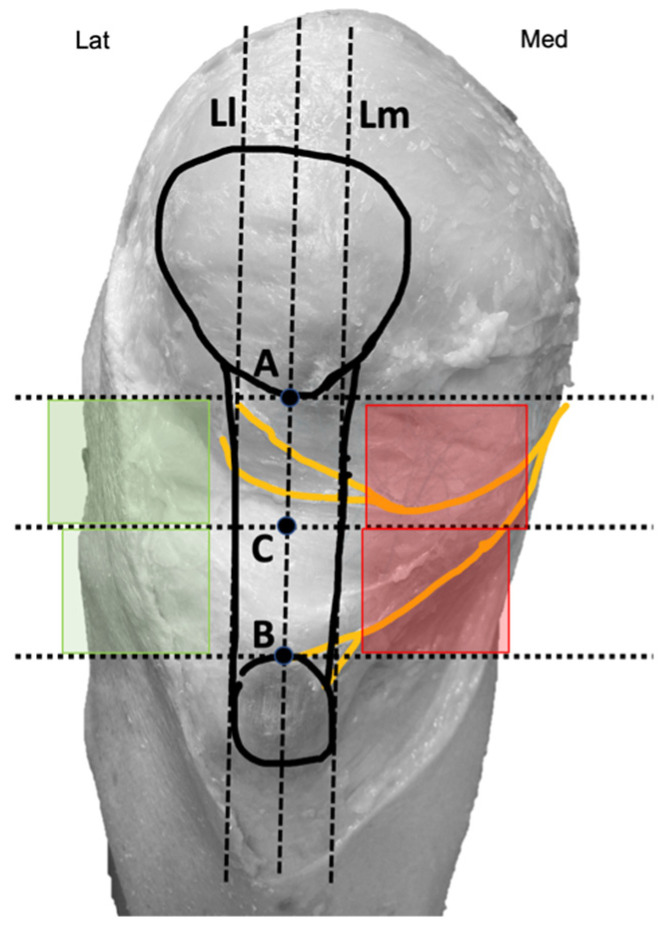
Diagram with reference points marked on the knee. Medial quadrants (red) and lateral quadrants (green).

Two bone reference points were marked on the knee. Point A was located at the inferior pole of the patella, while Point B was at the center of the superior border of the tibial tuberosity (Figure 2).

These points were intentionally chosen to ensure easy identification during therapeutic interventions. Finally, a third point (C) was identified as the midpoint between points A and B. A vertical line passing through all three points and the center of the patellar tendon was drawn, along with three horizontal lines intersecting each of the marked points. Additionally, two imaginary vertical lines, parallel and equidistant from the central line (L), were established: one aligning with the medial border of the patellar tendon (Lm) and the other with the lateral border of the patellar tendon (Ll) (Figure 2). Using these points and lines, four quadrants positioned above and below point C and parallel to the tendon were delineated: two medial quadrants (marked in red) and two lateral quadrants (marked in green) (Figure 2). These areas were represented as therapeutic windows because they coincide with the common access routes used by other authors for needle-based therapies in patellar tendinopathies [26,27,28,29,30].

### 2.4. The Needling Approach on the Fresh Cadaver

First, the needle approach to be used during the percutaneous electrolysis procedure was conducted on fresh cadavers. With the knee in flexion, an approach like the one used in clinical practice was simulated. Hence, the needle was inserted into the knee area from the lateral side, approximately 1 cm below the inferior pole of the patella and 1cm external to the Ll line (lateral border of the patellar tendon) targeting the deep interface of the Hoffa’s fat pad.

The same procedure was performed approximately 1 cm cranial to the tibial tuberosity and 1cm from the Ll line. All needle insertion procedures were performed with a 25 × 0.3 mm solid filiform needle (AguPunt, Barcelona, Spain).

### 2.5. The Percutaneous Electrolysis Procedure

A percutaneous electrolysis intervention targeting the interface between the patellar tendon and the Hoffa’s fat pad, both at the inferior pole of the patella and at the tibial tuberosity, was conducted in 10 healthy subjects. The intervention was ultrasound-guided using a Samsung^®^ HS50 ultrasound scanner equipped with a 14 MHz surface linear transducer (LA3-14AD, General Electrics, PRIM, Madrid, Spain). The procedures were performed by a physical therapist with 15 years of experience in ultrasound needling interventions. The ultrasound depth was set at 3 cm to ensure repeatability of the study.

**Figure 3:**
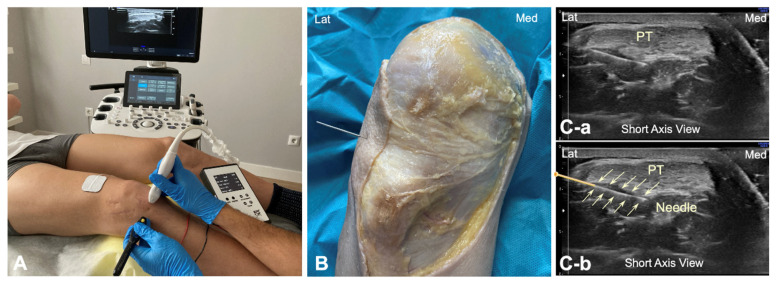
(**A**) Illustration of the percutaneous electrolysis approach at the interphase Hoffa’s fat pad—Patellar tendon (from lateral border); (**B**) Cadaver preparation with needle approach to the Hoffa’s fat pad interface from the lateral to medial side; (**C**) Ultrasound imaging (short axis view) of the needle reaching the interphase of the Hoffa’s fat pad without the needle marked (**C-a**) and with the needle marked (**C-b**). Lat (Lateral), Med (Medial), and PT (Patellar tendon).

To identify the infrapatellar nerve and its branches prior to puncture, long-axis and short-axis ultrasound imaging was conducted in the subjects in the same position as in the cadaveric study. A solid 25 × 0.3 mm filiform needle was then inserted into the deep interface of the patellar tendon from the lateral to the medial side, with the needle transversal to the patellar tendon (Figure 3A). The galvanic current dose used was 2 mA for 3 s. The tendon was targeted at the same point as in the cadaver model (Figure 3B). This approach was done under ultrasound guidance (Figure 3C).

## 3. Results

A total of 10 lower extremities from five cadaver specimens (2 males, mean age: 69 ± 4 years; 3 females, mean age: 75 ± 6 years) and 10 healthy volunteers (7 males, mean age: 42 ± 10 years old; 3 women, mean age: 33 ± 8 years) were included in the study.

Both the cadaveric and human model revealed a close anatomical relationship between the patellar tendon with infrapatellar nerve branches. In the cadaver model, the saphenous nerve was observed to emerge near the sartorius muscle, although showed some variability; in 10% of the cases, the nerve emerged anterior to the muscle, in 30% posterior to the muscle, and in 60% the nerve perforated the muscle. Thus, the infrapatellar nerve was divided into several branches. One infrapatellar branch with its collateral branches was directed towards the anterior knee, and another terminal branch was directed distally toward the leg. In fact, 70% of the cadavers had two infrapatellar branches, one superior and one inferior (Figure 1), while the remaining 30% had three branches.

After bifurcation, infrapatellar nerve branches ran subcutaneously obliquely from medial to anterior and lateral, crossing in front of the patellar tendon in a slightly concave manner (Figure 1). In all cadavers, the superior and inferior infrapatellar branches ran through the superior and inferior medial quadrants (in red in Figure 2). The branches crossed superficially to the patellar tendon, and only in 2 of the 10 knees they reached the lateral quadrants, specifically the superior lateral quadrant (in green in Figure 2). None of the branches reached the inferior lateral quadrant.

For the application of percutaneous electrolysis in the human model, an ultrasound-guided assessment was performed from the anteromedial and anterolateral side of the knee. Attempts were made to identify the infrapatellar nerve, and, in short axis of the tendon, it was only possible to visualize this branch in 2 out of 10 subjects (20%). In long axis, infrapatellar nerve branches were identified in 4/10 subjects at the level of the medial border of the patellar tendon at its distal insertion.

**Figure 4:**
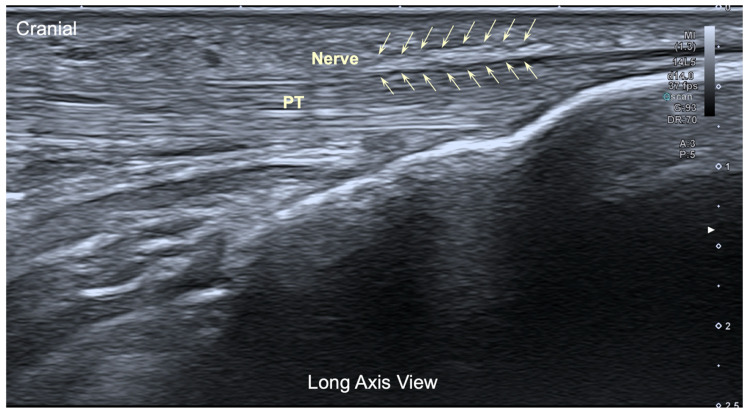
Ultrasound image (long axis view) of the infrapatellar nerve inferior branch. Pt: Patellar tendon.

The ultrasound image of the infrapatellar nerve found corresponded to a hyperechoic structure over the tendon (Figure 4), but in all cases, the localization was extremely difficult even for an experienced therapist.

## 4. Discussion

Cadaveric studies allow for better identification and assessment of therapeutic interventions with potential risks of damaging neurovascular tissues than other models. This study proposed an anatomical model targeting the interface of the patellar tendon and the Hoffa’s fat pad from a lateral-to-medial approach. The results also showed that no infrapatellar nerve injury occurred.

### 4.1. The Anatomy of Infrapatellar Nerve Branches

We visualized that infrapatellar nerve branches run from the medial to the anterior side of the knee, crossing the patellar tendon. It seems that the anatomical location of infrapatellar nerve branches can vary considerably between individuals and within both lower extremities of the same subject [31]. This anatomical variability in the formation and course of infrapatellar nerve branches was verified in our cadaver model since they ran close to the medial border and the anterior area of the patellar tendon, but the distance at which they bifurcated varied considerably. Our results are in line with previous cadaveric studies where differences in their emergence through the sartorius muscle, their branching, and their morphometry have been also described [23,32]. These changes in anatomical location make the infrapatellar nerve vulnerable to needling procedures applied through the anteromedial aspect of the knee. Iatrogenic injuries to this nerve have been described as a frequent complication in surgical knee interventions [19,20,21]. Injuries of the infrapatellar nerve cause a loss of sensitivity or paresthesia, and changes in surgical approaches to the knee have been proposed to avoid these complications [23,25].

Different studies have proposed the application of needle-biased approaches performed from the medial part of the knee [27,28,29,30,33]; however, the proposed anatomical model reveals that accessing through the superior or inferior medial area of the patellar tendon would represent a potential risk of infrapatellar nerve branch. Hence, the results of this cadaveric study suggest that the application of needling procedures from the medial knee area targeting the tendon–Hoffa’s fat pad interface can lead to a higher risk of injury for the infrapatellar nerve branches due to the complexity of visualizing these branches and the anatomical variability on their location. On the contrary, the application of needling approaches targeting the patellar tendon from the lateral edge of the knee can be successfully performed in all the volunteer subjects, without no nerve branch injury occurring in any of these needle insertions with galvanic electrical current application. Both the superior and inferior lateral quadrants of the knee are considered safe areas for needle insertion during these approaches.

### 4.2. Ultrasound-Guided Visualization for Needling Approaches

It is known that ultrasound imaging is an excellent tool for the study and evaluation of tendon pathology, but it is also considered an essential support in some needling interventions. In a cadaveric study, Abat et al. described that accuracy of ultrasound-guided injections targeting the patellar tendon interface was higher (74%) than non-ultrasound-guided injections (11%) [34]. The use of ultrasound imaging is also a reliable method to minimize the risk of injury by reducing the incidence of undesirable puncture of surrounding sensitive tissues in risky anatomical areas. In fact, needling approaches such as percutaneous electrolysis need appropriate identification of structures and real-life visualization of possible anatomical variations for a less risky application of the technique [35]. Arias-Buria et al. reported that ultrasound guidance improved accuracy of needling insertion into the patellar tendon as compared to palpation guidance [30]. Nevertheless, our study has shown that ultrasound visualization of the infrapatellar nerve branches is difficult, accordingly, this situation explains why it is essential to identify (with the cadaver model) safe therapeutic windows for clinical needle approaches. We do not know if the use of Power Doppler imaging could enhance visualization of the infrapatellar nerve branches in clinical practice.

### 4.3. The Management of Surrounding Tendon Structures

Regarding the management of patellar tendinopathies, it has been shown that the interface between the patellar tendon and the Hoffa fat pad is clinically relevant for the development of symptoms [36]. The periphery of the tendon is an important area where algogenic substances can be accumulated, leading to neovascularization and neoinnervation processes [37]. This situation would explain why current treatments for tendinopathies try to manage the peripheral area as well as intratendinous tissue [38]. Evidence suggests that percutaneous electrolysis targeting the interface and the intratendinous tendon area improves pain and functionality in individuals with patellar tendinopathy [14,15,16]. Accordingly, the anterolateral approach described in this cadaver model can be a viable and safe option for the management of patellar tendinopathy.

### 4.4. Limitations

Finally, some potential limitations of this theoretical model must be acknowledged. First, anatomical dissection was performed on just five cadavers. Since anatomical variability exists, studies including a higher number of cadavers, including both sex and covering different age ranges, could help to elucidate anatomical variability. Thus, the ultrasound-guided procedure was also conducted in just 10 subjects. Data on sex differences were not collected, and anthropometric data of the lower extremities could influence nerve anatomy. Second, we used two anatomical reference points because they are common sites for the management of tendinopathies. The middle portion of the patellar tendon area was not studied. Consequently, current data should be considered for the points addressed. Third, all needle insertions were performed by an experienced therapist. We do not know the safety of this needle procedure when performed by novice physical therapists. Finally, as a theoretical model, it should be implemented into clinical practice to determine its real applicability and effectiveness.

## 5. Conclusions

This study described a theoretical anatomical model targeting the interface of the patellar tendon and the Hoffa fat pad from a lateral-to-medial approach. The results support the use of a lateral approach, with ultrasound guidance when applied into real subjects, as a potentially safe approach to apply needling interventions targeting the patellar tendon. We observed that infrapatellar nerve branches are vulnerable to needle procedures applied through the anteromedial side of the knee.

## Data Availability

All data derived from this study are presented in the text.
